# Comparisons of Correlates of Viral Suppression Among Adults Living With HIV/AIDS in Tanzania: Analysis With and Without Including Survey Designs

**DOI:** 10.1155/arat/3817430

**Published:** 2025-02-18

**Authors:** Dayani Adam, Ramkumar T. Balan

**Affiliations:** ^1^Department of Social Sciences and Humanities, Mzumbe University—Mbeya Campus College, Mbeya, Tanzania; ^2^Department of Mathematics and Statistics, University of Dodoma, Dodoma, Tanzania

**Keywords:** antiretroviral therapy, complex survey design, design effects, viral load suppression

## Abstract

The effects of ignoring survey designs during the analysis of complex survey data may lead to biased estimates. This has been a common practice for most researchers. It is more critical for public health data which involve the clinical decisions that decide the fate of people's lives. This analysis compares the estimates of factors of viral load suppression (VLS) with and without including survey designs using the Tanzania HIV Impact Survey (THIS). This survey reveals factors associated with VLS among Tanzanians living with HIV/AIDS. The correlates of VLS were examined using multivariable logistic regression models in both cases with and without including survey design. The study unveils significant correlates such as age, middle wealth quintile, CD4, adherence, and antiretroviral (ARV) detection status of a patient. Furthermore, the study emphasizes the essence of properly accounting for CSD. Failure to do so may result in biased parameter estimates and incorrect variances; hence, incorrect inferences. Thus, the study's findings on VLS determinants have significant practical implications that allow government agencies and stakeholders to establish targeted and successful HIV/AIDS prevention and treatment initiatives. Consequently, this study suggests a complex design as an approach for obtaining unbiased estimates on the national representative surveys.

## 1. Introduction

Over the past 40 years, the global pandemic of the human immunodeficiency virus (HIV) has had a tremendous detrimental influence on public health [1]. According to Zenu et al. [2], viral infection has been estimated to have killed 36.3 million people since it first appeared and infected 79.3 million people globally. According to the most current estimate, 1.5 million new cases of HIV infection and 650,000 deaths from AIDS will occur by the end of 2021 [3]. Of the people living with HIV (PLHIV), 36.7 million (95.5%) are adults, while 1.7 million (4.5%) are young children between the ages of 0 and 14 [3, 4].

The Joint United Nations Programme on HIV/AIDS (UNAIDS) and other relevant stakeholders fighting epidemic emphasize treatment programmes because of the prevalence, consequences, and recurrence of new infections of HIV/AIDS [5, 6]. That is, the diagnosis of new HIV infections necessitates intensification of the use of antiretroviral therapy programs (ART) for the good health and well-being of the PLHIV. The use of ART, which has been widely accessible since the middle of the 1990s facilitates achievement of the UNAIDS targets of 95-95-95 [7, 8]. This target entails that to end the HIV epidemic by 2030, 95% of PLHIV should be diagnosed, 95% of those diagnosed should be linked to care and treatment, and 95% of those linked to care and treatment should achieve viral load suppression (VLS) [9]. The study by Melku et al. [10] asserts that VLS is an important indicator of the effectiveness of ART among HIV adult patients. This is because reducing viral loads is crucial for combating the disease in the future.

However, Tanzania has made progress in ART care, VLS levels are unsatisfactory in different age groups [11, 12]. In the same vein, Melku et al. [10] posit that most HIV patients experience unsuppressed viral load which is a significant predictor of patient mortality. For instance, Tanzania HIV Impact Survey (THIS) conducted in 2016–2017 shows that only 87.8% of HIV adult patients on ART were virally suppressed [13]. However, the THIS 2022-2023 report indicated an increase in VLS to approximately 94.3% among adult HIV patients [14]. Similarly, Mnvaza et al. [12] found that 88% of patients living with HIV/AIDS had VLS in the Kilombero and Ulanga districts. Again, a VLS rate of almost 85.3% was discovered in a different study by Charles et al. [15] who focused on children in susceptible groups. The unsatisfactory VLS levels show that Tanzania has to step up its efforts to improve HIV care and treatment.

Despite good progress of the programmatic achievements of the care and treatments in Tanzania, still, there is a need to obtain accurate and correct estimates for better monitoring and evaluation of the programmes. In this regard, accounting for the design of the survey during analysis has continued to be used and yields significant results. For instance, Workie et al. [16] included a survey design in the logistic regression on the determinants of unmet needs for family planning among women aged 15–49 years in Ethiopia. Also, the study by Abimiku et al. [17] on the factors associated with VLS in Nigeria found that young people and those with undetectable antiretrovirals (ARVs) had lower odds of VLS. Another study by Habyarimana and Ramroop [18] used a complex survey on the proportional hazard model on the determinants of malnutrition in Rwanda. The study found that birth order, mother's education, gender of the child, birth weight of the child, marital status of the mother, body mass index, anemia, multiple births, and whether or not the child had a fever before the survey were the factors associated with malnutrition in Rwanda. These studies utilized multistage sampling.

Thus, including the design of the surveys during the analysis was critical for robust estimates which most researchers tend to ignore. Nevertheless, misspecifications of the survey design, particularly omission of the second sampling stage, substantially affected both point estimates of health characteristics and their standard errors (SEs) [19]. From this study, it also indicated that lack of consideration of the sampling design only resulted in small differences in variance, while weighting significantly changed the point estimates for lifestyle factors. Since these data are nationally representative, multistage sampling is commonly employed, ensuring that target respondents are drawn from diverse population groups. Hence, consideration of the design in the analysis is imperative. These characteristics make the data an essential tool for assessing VLS among PLHIV.

Most of the studies carried out using binary or ordinal logistic regression did not consider the complex sampling design (CSD) [20, 21]. However, these studies used multistage sampling, which may influence the accuracy of estimates. Therefore, this study intends to show the comparison of the two estimates with and without accounting for the survey designs on assessing correlates of the VLS using THIS of 2022-23.

## 2. Materials and Methods

### 2.1. Study Participants

From THIS 2022-23, 33,663 participants aged 15 years and older were selected if they had participated in both interview and biomarker datasets of the survey [14]. These included those who tested positive for HIV during the survey. Only HIV-positive adults who received ART care were included among participants testing positive for HIV infection. Ultimately, the sample size of 1555 HIV-positive adults with VLS status was determined for this study.

### 2.2. Study Design and Sampling Methods

This analysis adopted a cross-sectional survey of THIS of 2022-2023 involving HIV adult patients receiving ART [22]. This survey used a two-stage stratified cluster design. This sampling design was taken from a Population-Based HIV Impact Assessment (PHIA) survey of 31 regions of Tanzania which were divided into 28 strata. In this study, 26 regions of Tanzania Mainland were treated as separate strata. In the case of Zanzibar, all five regions were combined into two strata, Pemba and Unguja, where Pemba comprises Kaskazini Pemba and Kusini Pemba and Unguja comprises Kusini Unguja, Mjini-Magharibi, and Kaskazini Unguja. The initial and the second stages of the sampling procedure were further separated. Enumeration areas (EAs), which accounted for around 567 of the sample in the first stage, were grouped into clusters as the primary sampling units. As the initial step, primary sample units were chosen using probabilities corresponding to the number of households based on the population census denominator from 2022.

The second stage of selection involved the probability proportional to size (PPS) method for the random selection of a sample of households from each selected EA. This clustering assumes that the population is grouped naturally and captures intracluster homogeneity and intercluster variability. This, in turn, further refines the SE estimates of the traditional methods by taking into account the intraclass correlations between individuals within the same cluster. Stratification was done by dividing the population into 28 well-defined national strata. This approach enriched the precision of the estimates but also allowed for regional differences in HIV prevalence and other health outcomes, such as access to ART. Eventually, stratification reduces sampling error and improves the representativeness of subgroups.

### 2.3. Sampling Weights

The current study incorporated jackknife replicate weights to ensure unbiased results and capture the complexities of the sampling design. These weights adjusted estimates for the differential probabilities of selection at each stage of sampling by including them in the analyses of survey data. These weights are from both the interview and biomarker datasets. The original weights are intwt0 for the interview data set and btwt0 for the biomarker dataset. The initial weights represent the basic design of the survey. There were 277 replicate weights for the interview dataset called from intwt001 to intwt277, and similarly, there were 277 replicate weights for the biomarker dataset, from btwt001 to btwt277. These replicate weights allow for accounting for variability and provide appropriate variance estimation in their SE estimates.

#### 2.3.1. Sources of Data

We applied the secondary data from the 2022-2023 THIS survey. This nationally representative survey was designed to collect data on the key biological endpoints to provide estimates of HIV risk, burden, and efficiency of ARTs in the treatment process. Two separate datasets, the individual and biomarker datasets, were found from the extracted datasets. These datasets were joined to form a single dataset that was utilized for analysis in this study.

### 2.4. Study Variables

#### 2.4.1. Outcome Variables

VLS was an outcome variable to determine whether the viral load had been suppressed to an undetectable level. If viral loads are less than 1000 copies per milliliter, they are considered suppressed; if they are larger than 1000 copies per millimeter implies the viral load is unsuppressed.

### 2.5. Predictor Variables

The predictor variables were categorized into sociodemographic, interpersonal, and treatment-related correlates. The sociodemographic variables include age, marital status, employment status, education level, sex, residential setting, and wealth quintile. Interpersonal variables are adherence, attitude, stigma, knowledge about HIV/AIDS, and Alcohol Use Disorders Identification Test (AUDIT_3C). On the other hand, the treatment-related variables include Class Differentiation 4 (CD4) counts, time between the initial diagnosis and the survey, antiretroviral medication detection status in the blood, and the types of ARV regimen employed. Indicators such as an understanding of how therapy can render HIV undetectable and its function in hesitant HIV transmission were used to gauge participants' knowledge of HIV/AIDS. Indicators such as the conviction that children with HIV should go to school, the readiness to purchase vegetables from an individual with HIV, and the sense of embarrassment if a family member had HIV were used to gauge attitudes toward the virus. Also, based on the stigma variable, indicators such as feeling embarrassed by disparaging remarks about HIV, being subjected to taunts and harassment, losing one's work, having to move because of one's HIV status, and being refused services because of one's status were included. AUDIT_3C for alcohol use disorders was calculated based on three indicators: frequency of alcohol use, typical alcohol consumption amount, and frequency of high alcohol consumption.

### 2.6. Ethical Considerations

This was an open-access website, and the data have been anonymized. Thus, the researcher did not access personally identifiable information such as file numbers when extracting the information. The researcher requested a research permit from the University of Dodoma. Also, the researcher followed THIS guidelines on data use provided by relevant authorities such as the Institutional Review Boards from the Center of Disease Control (CDC), Columbia University, Westat, Tanzania's National Institute for Medical Research (NIMR), and the Zanzibar Medical Research and Ethics Committee [23]. During THIS study, experts involved in the data collection such as laboratory technologists, nurses, interviewers, and supervisors all received training on the ethical protection of survey participants, acceptable clinical and laboratory practices, and data confidentiality agreements.

### 2.7. Data Analysis

The data were analyzed using the statistical analysis software (SAS) version 9.4. The analysis involved the use of weighted percentages and bar graphs for detailed information on the VLS status of HIV adult patients in Tanzania. Furthermore, the Rao–Scott chi-square test was used to determine the association between soci-demographic characteristics and VLS. The multivariable binary logistic regression models were employed to compare the correlates of the VLS by taking into account simple random sampling (SRS) and CSD. The multivariable binary logistic regression model was appropriate because the VLS was treated as the binary outcome. The model was designed to describe the probability of VLS in HIV adult patients. The multivariable binary logistic regression model based on SRS was used to model the probability of the VLS given the values of the predictor variables as indicated in equations in (1) and (2).

These predictor variables were modeled separately based on sociodemographic, interpersonal, and treatment-related factors as correlates of VLS. For each predictor variable and corresponding correlate (i.e., sociodemographic, interpersonal, and treatment-related factors), the analysis was conducted for each individual *i* = 1, 2,…, *n*.

The logit model is given by

(1) $$\begin{array}{c} \text{Logit }\left(\pi \right)=\beta_{0}+\beta_{1}x_{i1}+\cdots +\beta_{p}x_{\text{ip}}, \end{array}$$

where *π*_*i*_ is the probability that VLS achieved (*Y*_*i*_ = 1), *β*_0_ is the intercept parameter, *β*_*i*_(*i* = 1, 2,…, *p*) is the slope of the parameters, and *x*_1_ stands for the predictor variables. The expression on the left-hand side is the logit or log odds. The logit equation for the probability of the VLS is as follows:

(2) $$\begin{array}{c} \pi_{i}=\frac{\exp\left(\beta_{0}+\beta_{1}x_{i1}+\cdots +\beta_{p}x_{ip}\right)}{1+\exp\left(\beta_{0}+\beta_{1}x_{i1}+\cdots +\beta_{p}x_{ip}\right)}. \end{array}$$

Similarly, the multivariable binary logistic regression model was also adopted with the incorporation of CSD. CSD features including jackknife replicate weights, clustering, strata, and design effects (deff) were incorporated in the analysis. This model was also modeled with the incorporation of the CSD in a stage-cluster sampling design. Let *U* = (1, 2,…, *N*) be the finite HIV adult patient population which was divided into *h* = 1, 2,…, *H* regions as strata, and each stratum was further divided into *j* = 1, 2,…, *n*_*h*_. EAs as primary sampling units are constituted by *i* = 1, 2,…, *n*_*hj*_ households' secondary sampling unit. On the other hand, logistic regression with CSD can be written as follows:

(3) $$\begin{array}{c} \pi_{hji}=P\left(\frac{Y_{hji}=1}{X_{hji}}\right). \end{array}$$

Equation (3) is the probability of the VLS in the sample. The jackknife replicates weights in each sampling unit denoted by *w*_*hji*_ for *h*_*hji*_. The model in the form of the log of odds is given by the following equation:

(4) $$\begin{array}{c} \log\left(\frac{\pi_{hji}}{1-\pi_{hji}}\right)=X_{hji}^{\prime }\beta . \end{array}$$

In addition, the comparison of the correlates was done based on the adjusted odds ratio (aOR), SEs of the estimates, deff, and predictive accuracy of the model using concordance index and area under the curve (AUC). Deff is necessary for surveys to resolve difficulties from CSD. They calculate the variance ratio of a CSD to SRS, boosting accuracy and precision by modifying the sample size for cluster sampling. Deff accounts for intraclass correlation within clusters, with values greater than one (deff > 1) indicating greater variance than SRS and values less than one (deff < 1) indicating less variance. When CSD variance meets SRS, deff equals one (deff = 1), serving as a reference point. Scaling estimators with the square root of deff is a popular practice.

## 3. Results and Discussion

**Figure 1:**
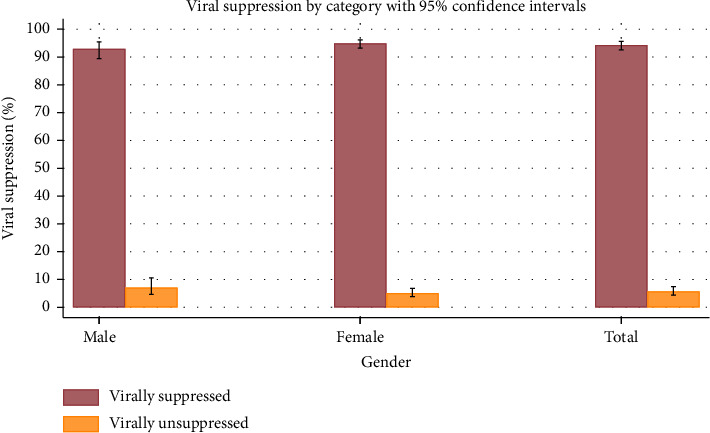
Viral suppression status by sex.

The study reported that 1469 (94.3%) of the HIV adult patients who received ART had VLS, while 86 (5.7%) did not. Furthermore, it was found that 1054 (94.9%) of the HIV-positive female patients receiving ART had VLS compared to 56 (5.1%) who did not. For HIV-positive male patients, 415 (92.9%) had VLS, while 30 (7.1%) had undetected viral loads.  Figure 1 shows the survey respondents' VLS by gender.

We identified that marital status and age of respondents were associated with VLS. The study also discovered that different sociodemographic factors affected VLS rates differently. Males were suppressed at a rate of 92.9% (95% CI: 89.4–95.4), whereas females were suppressed at 94.9% (95% CI: 93.2–96.2). Higher VLS rates were seen among people with lower education levels, revealing a trend in educational attainment. The rates of VLS were only slightly influenced by marital status and home location. The VLS rate was the highest for people aged 65 years and above 96.9% (95% CI: 90.6–99.1) and the lowest for people aged 15 to 24 years 84.8% (95% CI:72.4–92.3). Quintiles of wealth also revealed differences, with the middle quintile having the highest VLS 97.4% (95% CI: 95.0–98.7). Employment status had a small bearing; those employed had a greater suppression rate of 95.3% (95% CI: 92.9–96.9) than those unemployed 93.4% (95% CI: 90.8–95.3).  Table 1 indicates the information on the sociodemographic characteristics by the VLS status.

In this study, the probabilities of VLS of the HIV adult patients were modeled as the functions of sociodemographic, interpersonal, and treatment-related factors. Based on the models with SRS, Hosmer and Lemeshow test for sociodemographic (*χ*^2^ = 9.51, *p* = 0.3013), interpersonal (*χ*^2^ = 4.14, *p* = 0.2466), and treatment-related (*χ*^2^ = 3.56, *p* = 0.2511) which all models adequately represented the data. The predictive accuracy of the model was done by using the AUC of receiver operating characteristics (AUC-ROC) curve. The AUC-ROC of the sociodemographic, interpersonal, and treatment-related correlates were 0.6972, 0.5695, and 0.8305, respectively. The logistic regression models with CSD produced significant results in all domains according to the likelihood ratio tests: treatment-related factors were 19098.6 (*p* < 0.0001), interpersonal factors were 1416.63 (*p* < 0.0001), and sociodemographic factors were 4065.02 (*p*  <  0.0001). The models' predictive accuracy was indicated by the c-statistics, which were 0.811 for treatment-related correlates, 0.557 for interpersonal factors, and 0.665 for sociodemographic factors. With treatment-related factors showing the highest predictive accuracy, these figures show that the models exhibited predictive accuracies of 66.5%, 55.7%, and 81.1%, respectively. The logistic regression model for treatment-related correlates has the highest predictive accuracy. The goodness of fit and predictive accuracy of the model is presented in Table 2.

Table 3 reveals several significant correlates associated with VLS using CSD. Among sociodemographic factors, age was a key determinant. Compared to individuals aged 15–24 years, those aged 35–44 years had significantly higher odds of achieving VLS (aOR: 3.10, 95% CI: 1.20–7.90, *p* = 0.021). Similarly, individuals aged 45–54 years exhibited increased odds of VLS (aOR: 6.50, 95% CI: 2.27–18.52, *p* = 0.001). Older individuals, specifically those aged 55–64 years and 65 years or older, also showed significantly higher odds, with aORs of 3.78 (95% CI: 1.11–12.87, *p* = 0.035) and 6.53 (95% CI: 1.66–25.76, *p* = 0.009), respectively. Wealth status also influenced VLS, with individuals in the middle wealth quintile being significantly more likely to achieve VLS compared to those in the lowest quintile (aOR: 3.50, 95% CI: 1.41–8.52, *p* = 0.009). Treatment adherence was strongly associated with VLS, with individuals who adhered to treatment having an aOR of 2.67 (95% CI: 1.26–5.64, *p* = 0.012). The results of the analysis showed that there was a difference in the significance of HIV treatment knowledge between SRS and CSD models; it was not significant in CSD (aOR: 1.50, 95% CI: 0.95–2.43, *p* = 0.076) but significant in SRS (aOR: 1.60, 95% CI: 1.01–2.48, *p* = 0.047). Treatment-related factors were also highly significant. Individuals with a CD4 count above 500 were more likely to achieve VLS (aOR: 2.72, 95% CI: 1.18–6.27, *p* = 0.021). The presence of detectable ARVs was the most strongly associated factor, with an aOR of 37.6 (95% CI: 15.68–90.32, *p* < 0.0001). In addition, being on treatment for one or more years was associated with higher odds of VLS (aOR: 4.60, 95% CI: 1.75–11.86, *p* = 0.003).

The correlates in Table 4 reveal the CSD effects on SEs. The age groups had the highest deff for those aged 55–64 years, deff = 1.8007 and deft = 1.3419, which means that there was a large increase in SEs under CSD, whereas the 65+ group had a small effect, deff = 1.1075 and deft = 1.0534. For the wealth quintiles, the middle quintile had a slight increase in SEs, deff = 1.0719 and deft = 1.0353, which is 7.19% larger than that for SRS. Knowledge about HIV showed minimal impact (deff = 1.04 and deft = 1.1566), whereas treatment adherence displayed a moderate increase in SEs (deff = 1.2934 and deft = 1.1373), reflecting a 29.34% larger error under CSD. A CD4 level of 500 or above exhibited a strong influence (deff = 1.4067 and deft = 1.8600), while ARV detection status, with the reference group being showed a moderate impact (deff = 1.2876 and deft = 1.1347). Time since diagnosis of 1 year or more relative to less than a year also demonstrated a moderate increase in SEs (deff = 1.2942 and deft = 1.1376), indicating a 29.42% larger error under CSD. These findings underscore the variable influence of CSD across predictors, with the most pronounced effects observed for age groups and CD4 levels.

## 4. Discussion

We compared the correlates of VLS among adults living with HIV/AIDS in Tanzania using binary logistic regression models with and without CSD. The results showed that the models with CSD had higher SEs than those with SRS. This depicts that the precision of estimates depends on the type of survey design considered. By incorporating CSD, we captured the underlying structure of the data, leading to more accurate variance estimates, whereas neglecting CSD can lead to misleading conclusions and underestimate the true variability in the data. This is consistent with the findings by Habyarimana and Ramroop [18], Lumley and Scott [24], and Yirga et al. [25] reported that failure to account for CSD may result in biased estimates of SEs. As a result, some predictors considered to be significant in the SRS models may not reach significance when CSD is applied. For example, knowledge regarding HIV was significant in the SRS model but lost its significance in the CSD model.

Our study found that factors related to treatment, such as ART detection, CD4 levels, and time from diagnosis to the survey, were more strongly associated with VLS than interpersonal and sociodemographic factors. These findings reiterate that treatment-related factors are more important and need to be prioritized in VLS interventions, as established in studies by Mogosetsi et al. [26]. ART adherence emerged as a determinant of VLS, necessitating the implementation of targeted interventions to enhance medication adherence. Interventions aimed at improving access to ART, ART counseling, and psychosocial support will go a long way toward minimizing drug resistance and optimizing health outcomes. This observation is consistent with previous reports by Habte et al. [27] and Anito et al. [28] though there are reports of no association found in a study by McCarthy et al. [29] because of potential variations in both study populations and measurement methods.

Other important predictors of VLS were the presence of ARVs in blood specimens, reflecting treatment adherence. This calls for the need to monitor adherence within public health programs to improve outcomes for VLS, reduce transmission risks, and enhance the quality of life for PLHIV. These results are supported by studies that use CSD analysis, according to Abimiku et al. [17] and by Atnafu et al. [23], which also identified treatment-related factors as predictors of VLS.

CD4 count levels were significantly associated with VLS, indicating that viral suppression can be achieved regardless of immunological status. This finding reinforces the role of regular CD4 count monitoring as a component of comprehensive HIV care, consistent with studies from South Africa and elsewhere [30–32]. Incorporating CD4 counts into routine clinical practice may optimize ART regimens and improve patient outcomes.

The study did not find a significant association between VLS and female HIV-positive patients on ART. However, differences in behavioral patterns, adherence, and sociocultural dynamics might explain the lack of statistical significance. These findings align with studies by Abimiku et al. [17] but contrast with Atnafu et al. [23], who reported no association between gender and VLS in CSD models. Addressing gender-specific barriers to VLS could improve health outcomes and inform targeted public health strategies.

In addition, employment status in our analysis was not associated with VLS. This means that employment interventions may be mounted but would have no direct effects on VLS; they have to be incorporated into more holistic initiatives that tackle social determinants of health. These findings are in agreement with the studies by Habte et al. [27] and Anito et al. [28], although in contrast, McCarthy et al. [29] and Okere et al. [31] reported significant associations.

The significant inclusion of CSD provided evidence that the results have higher deffs and robustness in SE estimates. Indeed, this confirms that not accounting for CSD in studies will certainly lead to underestimated SEs and incorrect inferences regarding the significance of predictors [33, 34]. Such findings strengthen the call for the incorporation of CSD in health research studies to enhance the validity and reliability of estimates.

Also, the CSD model is higher in c-index and AUC values than SRS. Thus, it underlines the additional value of models with CSD in assessing public health data to make sure that findings are representative and accurate for interventions.

## 5. Conclusion

The study identified the middle wealth quintile, age, CD4 counts, adherence, ARV detection, and time since initial diagnosis as key correlates associated with VLS among adults living with HIV. These factors consistently increased the chances of attaining VLS, thus providing practical data for personalizing medical interventions to increase VLS rates and improve health outcomes in certain subpopulations.

Results highlight that analyses need to consider CSD. While this will lead to higher SEs for some correlates, using the CSD enhances statistical estimate reliability and protects against biased conclusions. Lack of consideration of CSD at the time of analyses will produce erroneous estimates, which can compromise the effectiveness of potential public health interventions.

Researchers and policy-makers are also recommended to consider deff in the choice of analytical approaches. Good practice in survey design methods at the outset improves the evidence, enhances the quality of public health data, and enables less biased decision-making. Ignoring this sound methodology risks compromising interventions and their public health effects.

## 6. Area for Further Study

Further studies are recommended using longitudinal data analysis to compare the time-varying factors affecting ART adherence and VLS in both the CSD and SRS models for insight into targeted interventions for sustained VLS in HIV patients.

## Figures and Tables

**Table 1:** Weighted percentages by viral load suppression status.

Variable	Suppressed (*n* = 1469)		Unsuppressed (*n* = 86)		*p* value
	*n*	Weighted% (95% CI)	*n*	Weighted% (95% CI)	
Sex					
Male	415	92.9 (89.4–95.4)	30	7.1 (4.6–10.6)	0.1635
Female	1054	94.9 (93.2–96.2)	56	5.1 (3.8–6.8)	
Education					
No education	245	95.3 (91.1–97.6)	13	4.6 (2.4–8.9)	0.3937
Primary	1045	94.4 (92.3–95.9)	58	5.6 (4.1–7.7)	
Secondary	163	91.7 (86.2–95.2)	15	8.3 (4.8–13.7)	
More than secondary	13				
Marital status					
Never married	128	88.5 (81.7–93.0)	19	11.5 (7.0–18.3)	**0.0166**
Married/living together	751	94.3 (91.7–96.1)	44	5.7 (3.9–8.3)	
Divorced	307	95.7 (92.4–97.6)	14	4.3 (2.4–7.6)	
Widowed	281	96.4 (92.3–98.4)	9	3.6 (1.6–7.7)	
Residential setting					
Urban	531	94.1 (90.9–96.1)	33	5.9 (3.8–9.0)	0.8104
Rural	938	86.8 (83.1–90.5)	53	5.6 (4.0–7.6)	
Age					
15–24	64	84.8 (72.4–92.3)	13	15.2 (7.7–27.6)	**0.0009**
25–34	243	90.6 (85.6–93.9)	25	9.4 (6.1–14.4)	
35–44	416	94.6 (91.1–96.8)	23	5.4 (3.2–8.9)	
45–54	435	97.2 (94.6–98.6)	14	2.8 (1.4–5.4)	
55–64	208	95.5 (89.2–98.2)	7	4.5 (1.8–10.8)	
65+	103	96.9 (90.6–99.1)	4	3.0 (0.9–9.4)	
Wealth quintile					
Lowest	255	92.4 (87.6–95.4)	21	7.6 (4.6–12.4)	0.1770
Second	364	93.6 (89.4–96.2)	21	6.4 (3.8–10.6)	
Middle	384	97.4 (95.0–98.7)	12	2.6 (1.3–5.0)	
Fourth	300	93.9 (90.0–96.4)	21	6.0 (3.6–9.9)	
Highest	166	93.9 (88.7–96.9)	11	6.1 (3.1–11.3)	
Employment					
Employed	682	95.3 (92.9–96.9)	35	4.7 (3.1–7.0)	0.1878
Not employed	787	93.4 (90.8–95.3)	86	6.6 (4.7–7.4)	

*Note:* Bold values represent statistical significance at *p* < 0.05.

**Table 2:** Goodness of fit and predictive accuracy of the model with CSD.

Relationship between predicted probability and observed responses				Likelihood ratio test
*Sociodemographic correlates*				
Percent concordant	65.5	Somer'D	0.330	*χ* ^2^ = 4065.02 ***p* < 0.001**
Percent discordant	32.5	Gamma	0.337	
Percent tied	2.0	Tau-a	0.035	
Pairs	125904	c	0.665	

*Interpersonal correlates*				*χ* ^2^ = 1416.63 ***p* < 0.0001**
Percent concordant	41.0	Somer'D	0.115	
Percent discordant	29.5	Gamma	0.163	
Percent tied	29.2	Tau-a	0.012	
Pairs	126334	c	0.557	

*Treatment-related correlates*				*χ* ^2^ = 19098.6 ***p* < 0.0001**
Percent concordant	75.3	Somer'D	0.622	
Percent discordant	13.1	Gamma	0.703	
Percent tied	11.6	Tau-a	0.050	
Pairs	56800	c	0.811	

*Note:* Bold values represent statistically significant at *p* < 0.05.

**Table 3:** Comparison of the correlates based on SRS and CSD.

Variable	SRS			CSD		
	aOR (95% CI)	Std. error	*p* value	aOR (95% CI)	Std. error	*p* value
*Sociodemographic*						
Sex (ref = male)						
Female	1.57 (0.94–2.59)	0.403	0.082	1.59 (0.93–2.66)	0.3957	0.074
Age (ref = 15–24)						
25–34	1.57 (0.70–3.49)	0.6408	0.272	1.55 (0.63–3.80)	0.6752	0.320
35–44	2.85 (1.24–6.59)	1.220	**0.014**	3.10 (1.20–7.90)	1.4080	**0.021**
45–54	5.00 (2.01–12.41)	2.32	**0.001**	6.5 (2.27–18.52)	3.303	**0.001**
55–64	4.61 (1.55–13.71)	2.56	**0.006**	3.78 (1.109–12.87)	2.2491	**0.035**
65+	4.59 (1.28–16.51)	2.99	**0.020**	6.53 (1.66–25.76)	4.3521	**0.009**
Residential setting (ref = rural)						
Urban	0.99 (0.48–1.79)	0.310	0.991	0.85 (0.36–2.77)	0.4357	0.710
Marital status (ref = never married)						
Married	1.69 (0.88–3.26)	0.5959	0.111	1.32 (0.54–3.22)	0.5723	0.528
Divorced	2.01 (0.91–4.48)	0.8208	0.08	1.62 (0.56–4.63)	0.8259	0.357
Widowed	2.10 (0.79–5.39)	1.005	0.142	1.47 (0.38–5.61)	0.9550	0.563
Wealth quintile (ref = first)						
Second	1.47 (0.771–2.800)	0.4834	0.242	1.25 (0.59–2.65)	0.4560	0.533
Middle	2.800 (1.311–5.98)	0.108	**0.008**	3.5 (1.41–8.52)	1.5140	**0.009**
Fourth	1.28 (0.59–2.72)	0.4938	0.530	1.62 (0.64–4.10)	0.7307	0.296
Fifth	1.44 (0.55–3.72)	0.6981	0.457	1.57 (0.48–5.07)	0.8937	0.438
Employment (ref = not employed)						
Employed	1.36 (0.86–2.15)	0.3179	0.185	0.85 (0.36–2.77)	0.435	0.124
Education (ref = no education)						
Primary	0.93 (0.48–1.79)	0.3107	0.823	1.12 (0.59–2.14)	0.3273	0.72
Secondary	0.87 (0.36–2.10)	0.3899	0.762	0.72 (0.29–1.77)	0.4594	0.47

*Interpersonal correlates*						
AUDIT_3C (ref = not)						
At risk	0.9 (0.42–1.94)	0.4322	0.796	0.69 (0.28–1.73)	0.3083	0.417
Attitude (ref = low)						
High	1.73 (0.89–3.37)	0.5872	0.106	1.59 (0.75–3.39)	0.5836	0.217
Knowledge (ref = low)						
High	1.6 (1.01–2.48)	0.3634	**0.047**	1.5 (0.95–2.433)	0.3464	0.076
Stigma (ref = high)						
Low	1.2 (0.37–3.88)	0.7156	0.790	1.64 (0.28–9.65)	1.4114	0.570
Adherence (ref = low)						
High	2.57 (1.373–4.802)	0.8200	**0.003**	2.67 (1.26–5.64)	0.9689	**0.012**

*Treatment-related correlates*						
CD4 (ref = less than 500						
More than 500	2.55 (1.34–4.83)	0.8317	**0.004**	2.72 (1.18–6.27)	1.103	**0.021**
ARVs (ref = not detected)						
Detected	40.4 (19.6–83.16)	14.88	**< 0.001**	37.6 (15.68–90.32)	15.99	**< 0.001**
Efavirenz (ref = no)						
Yes	0.06 (0.01–0.652)	0.0729	**0.018**	0.27 (0.03–2.48)	0.2828	0.229
Time (ref = less than a year)						
One or more than a year	5.3 (2.55–11.09)	1.9937	**< 0.001**	4.6 (1.75–11.86)	2.11	**0.003**

*Note:* Bold values represent statistical significance at *p* < 0.05.

**Table 4:** Design effects and design factor.

Variable	Coef.	Jack std. err.	Deff	Deft
Age (ref = 15–24)				
35–44	1.1011	0.4586	1.5770	1.2558
45–54	1.8395	0.5095	1.5528	1.2461
55–64	1.3004	0.5943	1.8007	1.3419
65+	1.8462	0.6656	1.1075	1.0534
Wealth quintile (ref = first)				
Middle	1.2269	0.4368	1.0719	1.0353
Knowledge (ref = low)				
High	1.468	0.679	1.04	1.1566
Adherence (ref = low)				
High	0.9812	0.3633	1.2934	1.1373
CD4 (ref = less than 500)				
More than 500	1.002	0.4049	1.4067	1.8600
ARVs (ref = not detected)				
Detected	3.627	0.4251	1.2876	1.1347
Time diagnosis (ref = less than a year)				
One or more than a year	1.5181	0.4639	1.2942	1.1376

## Data Availability

The data that support the findings of this study are available from the Population-Based HIV Impact Assessment. Restrictions apply to the availability of these data, which were used under license for this study. Data are available from https://phia-data.icap.columbia.edu/datasets?country_id=10&year_id=2022.

## References

[1] KFF The Global HIV/AIDS Epidemic. https://www.kff.org/global-health-policy/fact-sheet/the-global-hivaids-epidemic/.

[2] Zenu S., Tesema T., Reshad M., Abebe E. (2021). Determinants of First-Line Antiretroviral Treatment Failure Among Adult Patients on Treatment in Mettu Karl Specialized Hospital, South West Ethiopia; a Case Control Study. *PLoS One*.

[3] UNAIDS (2021). Fact Sheet—World AIDS Day 2021 Global. https://www.unaids.org/en/resources/fact-sheet.

[4] WHO HIV and AIDS. https://www.who.int/news-room/fact-sheets/detail/hiv-aids.

[5] Bekker L. G., Alleyne G., Baral S. (2020). Advancing Global Health and Strengthening the HIV Response in the Era of the Sustainable Development Goals: The International AIDS Society. *Lancet Comm*.

[6] Dybul M., Attoye T., Baptiste S. (2021). The Case for an HIV Cure and How to Get There. *Lancet HIV*.

[7] Zhao F., Benedikt C., Wilson D. (2020). Tackling the World’s Fastest-Growing HIV Epidemic.

[8] Xu J. J., Han M. J., Jiang Y. J. (2021). Prevention and Control of HIV/AIDS in China: Lessons From the Past Three Decades. *Chinese Medical Journal*.

[9] UNAIDS (2015). Understanding Fast-Track Targets. *Accelerating Action to End the AIDS Epidemic by 2030*.

[10] Melku M., Abebe G., Teketel A. (2020). Immunological Status and Virological Suppression Among HIV-Infected Adults on Highly Active Antiretroviral Therapy. *Environmental Health and Preventive Medicine*.

[11] Mchomvu R. D., Hussein A. K., Matee M. (2022). Determinants of Viral Load Non-Suppression Among HIV-Positive Children and Adolescents Attending Care and Treatment Clinics in Tabora Region, Tanzania. *Bulletin of the National Research Centre*.

[12] Mnzava D., Okuma J., Ndege R. (2022). Decentralization of Viral Load Testing to Improve HIV Care and Treatment Cascade in Rural Tanzania: Data From the Kilombero and Ulanga Antiretroviral Cohort. *Research Square*.

[13] TPHIA (2018). Tanzania Population-Based HIV Impact Assessment-Final Report. https://phia.icap.columbia.edu/wp-content/uploads/2019/06/FINAL_THIS-2016-2017_Final-Report__06.21.19_for-web_TS.pdf.

[14] PHIA (2023). Tanzania HIV Impact Survey. *National Bureau of Statistics (Tanzania)*.

[15] Charles J., Exavery A., Ally A. (2022). Rates and Determinants of Retention on ART Among Orphans and Vulnerable Children Living With HIV in Tanzania. *Frontiers in Public Health*.

[16] Workie D. L., Zike D. T., Fenta H. M., Mekonnen M. A. (2017). A Binary Logistic Regression Model With Complex Sampling Design of Unmet Need for Family Planning Among All Women Aged (15–49) in Ethiopia. *African Health Sciences*.

[17] Abimiku A. L., Ramadhani H. O., Moloney M. (2023). Factors Associated With Viral Suppression Among Adults Living with HIV on Antiretroviral Therapy in Nigeria: Analysis of a Population-Based Survey, 2018. *HIV Medicine*.

[18] Habyarimana F., Ramroop S. (2014). A Proportional Odds Model with Complex Sampling Design to Identify Key Determinants of Malnutrition of Children Under Five Years in Rwanda A Proportional Odds Model With Complex Sampling Design to Identify Key Determinants of Malnutrition of Children Unde. *Mediterranean Journal of Social Sciences*.

[19] Schmidt C. O., Alte D., Völzke H., Sauer S., Friedrich N., Valliant R. (2011). Partial Misspecification of Survey Design Features Sufficed to Severely Bias Estimates of Health-Related Outcomes. *Journal of Clinical Epidemiology*.

[20] Adeleye A., Oluwatomi A., Stephanie C O., Obiageri E E., Nnenna Ann U. (2021). Sexual Behaviors and HIV Status: A Population-Based Study Among Youths and Adults in Tanzania. *Journal of Infectious Diseases and Epidemiology*.

[21] Ikwara E. A., Atwijukiire H., Atuhaire R. (2024). Coverage and Factors Associated With Health Insurance Utilization Among Reproductive Women: Insights From Tanzania Demographic Health Survey 2022. *A Quantitative Study*.

[22] TPHIA (2023). Sampling and Weighting Technical Report. https://www.nbs.go.tz/statistics/topic/the-tanzania-hiv-impact-survey.

[23] Atnafu G. T., Moges N. A., Wubie M., Gedif G. (2022). Incidence and Predictors of Viral Load Suppression After Enhanced Adherence Counseling Among HIV-Positive Adults in West Gojjam Zone, Amhara Region, Ethiopia. *Infection and Drug Resistance*.

[24] Lumley T., Scott A. (2017). Fitting Regression Models to Survey Data. *Statistical Science*.

[25] Yirga A. A., Melesse S. F., Ayele D. G., Mwambi H. (2019). The Use of Complex Survey Design Models to Identify Determinants of Malnutrition in Ethiopia. *Journal of Human Ecology*.

[26] Mogosetsi N. J., Mabuza L. H., Ogunbanjo G. A. (2018). The Prevalence of HIV Load Suppression and Related Factors Among Patients on ART at Phedisong 4 Clinic, Pretoria, South Africa. *The Open Public Health Journal*.

[27] Habte T. M., Bondo C., Nkombua L. (2020). Association Between Social Support and Viral Load in Adults on Highly Active Antiretroviral Therapy—Witbank, South Africa. *South African Family Practice*.

[28] Anito A. A., Lenjebo T. L., Woticha E., Solomon F. (2022). Magnitude of Viral Load Suppression and Associated Factors Among Clients on Antiretroviral Therapy in Public Hospitals of Hawassa City Administration, Ethiopia. *HIV*.

[29] McCarthy M., Tao J., Lerebours A., Rodriguez C., Flanigan T. P., Sanchez M. C. (2022). Evaluating Barriers to Viral Suppression Among People With HIV in Santiago, Dominican Republic. *Journal of the International Association of Physicians in AIDS Care*.

[30] Rangarajan S., Colby D. J., Bui D. D. (2016). Factors Associated with HIV Viral Load Suppression on Antiretroviral Therapy in Vietnam. *Journal of Virus Eradication*.

[31] Okere N. E., Censi V., Machibya C. (2022). Beyond Viral Suppression: Quality of Life Among Stable ART Clients in a Differentiated Service Delivery Intervention in Tanzania. *Quality of Life Research*.

[32] Elashi B. A. Y., Van Wyk B. E. (2022). Factors Associated With Viral Suppression Among Adolescents on Antiretroviral Therapy in Free State Province, South Africa. *Southern African Journal of HIV Medicine*.

[33] Dey D., Haque S., Islam M., Aishi U. I., Shammy S. S. (2025). The Proper Application of Logistic Regression Model in Complex Survey Data: A Systematic Review. *BMC Medical Research Methodology*.

[34] Wirth K. E., Tchetgen E. J. T. (2014). Accounting for Selection Bias in Association Studies With Complex Survey Data. *Epidemiology*.

